# Supplementary material for: Therapeutic benefits of maintaining CDK4/6 inhibitors and incorporating CDK2 inhibitors beyond progression in breast cancer
Source: eLife. 2025 Dec 29;14:RP104545. doi: 10.7554/eLife.104545 (PMC12747521; doi:10.7554/eLife.104545)
Supplement: Figure 1—figure supplement 3—source data 1. [file elife-104545-fig1-figsupp3-data1.zip › Figure 1, figure supplement 3, source data 1/Figure 1, figure supplement 3, source data 1.pdf]

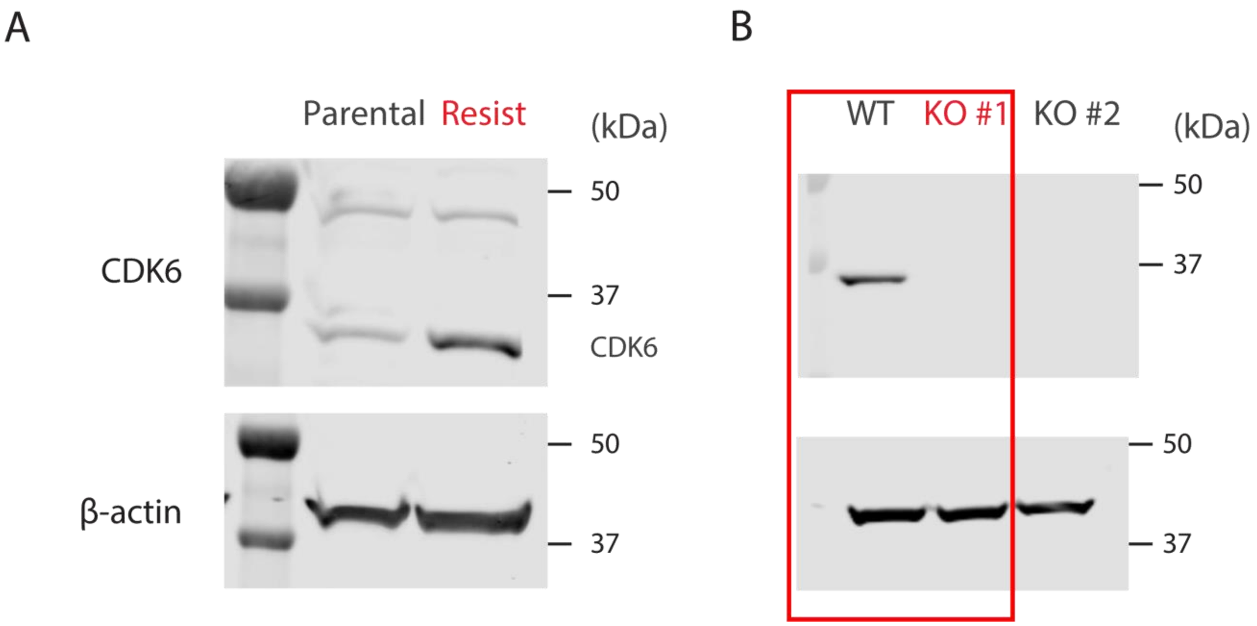

Figure 1, figure supplement 3, source data 1. Original membranes corresponding to Figure1 – figure supplement 3A, B. Immunoblot showing CDK6 and β-actin expression in drug-naïve and palbociclib-resistant cells (A) and WT and CDK6-KO cells (B). KO clone #1 was used for subsequent experimentation. Precision plus protein standards were used and molecular weights indicated.
