# Supplementary figures and images for: Therapeutic benefits of maintaining CDK4/6 inhibitors and incorporating CDK2 inhibitors beyond progression in breast cancer
Source: eLife. 2025 Dec 29;14:RP104545. doi: 10.7554/eLife.104545 (PMC12747521; doi:10.7554/eLife.104545)

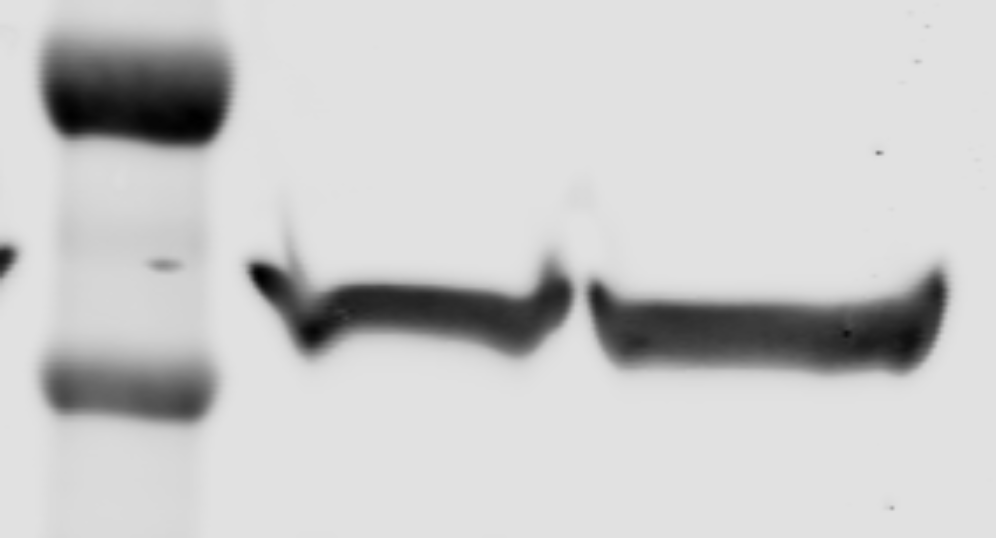

Supplement: Figure 1—figure supplement 3—source data 2. [file elife-104545-fig1-figsupp3-data2.zip › Figure 1, figure supplement 3, source data 2/panel A b-actin.png]

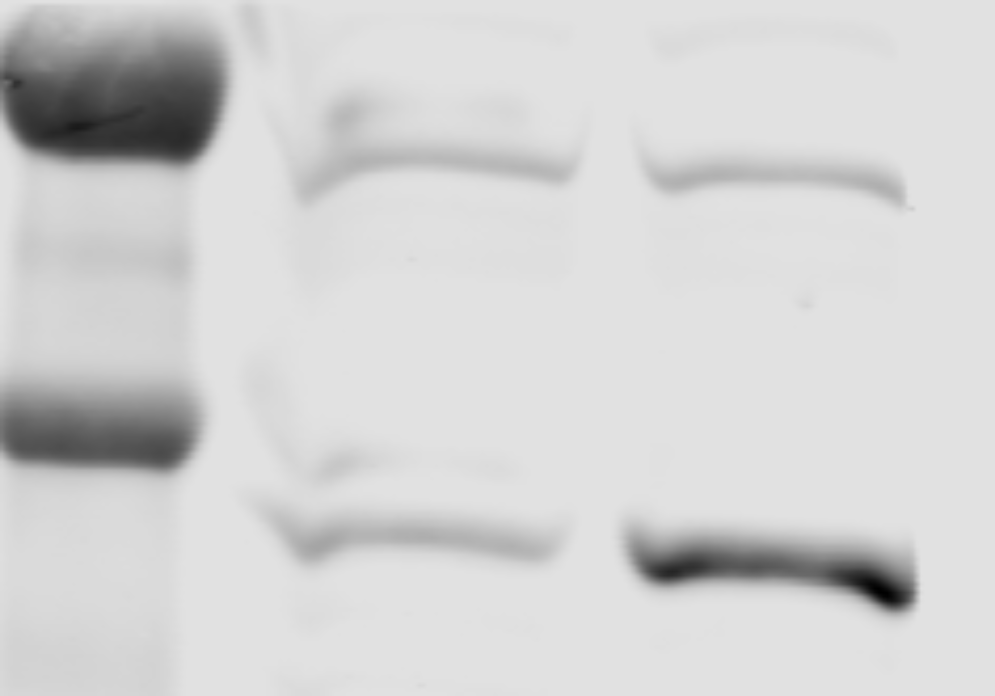

Supplement: Figure 1—figure supplement 3—source data 2. [file elife-104545-fig1-figsupp3-data2.zip › Figure 1, figure supplement 3, source data 2/panel A CDK6.png]

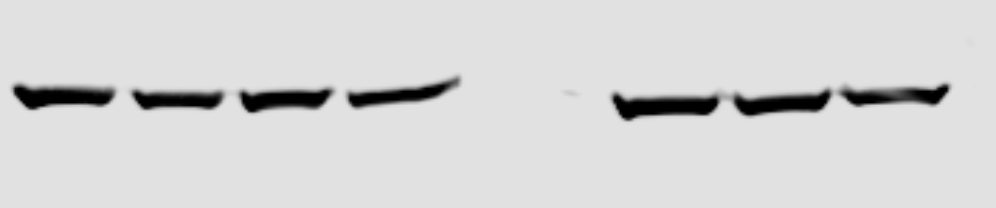

Supplement: Figure 1—figure supplement 3—source data 2. [file elife-104545-fig1-figsupp3-data2.zip › Figure 1, figure supplement 3, source data 2/panel B b-actin.png]

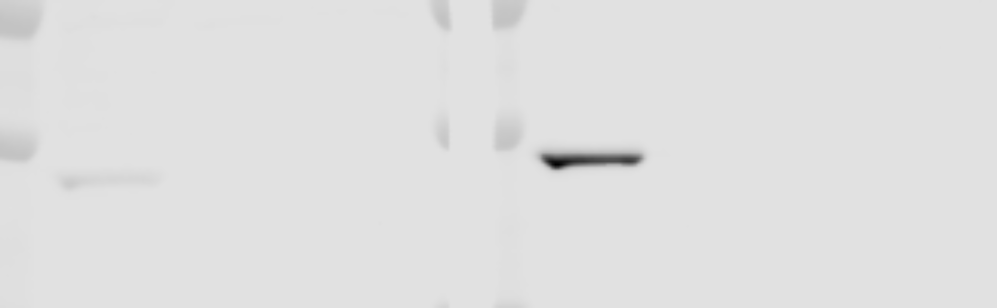

Supplement: Figure 1—figure supplement 3—source data 2. [file elife-104545-fig1-figsupp3-data2.zip › Figure 1, figure supplement 3, source data 2/panel B CDK6.png]

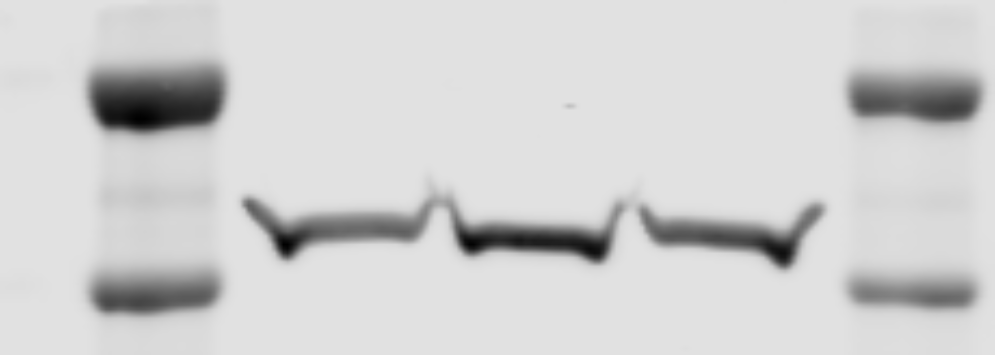

Supplement: Figure 3—source data 2. [file elife-104545-fig3-data2.zip › Figure 3, source data 2/b-actin.png]

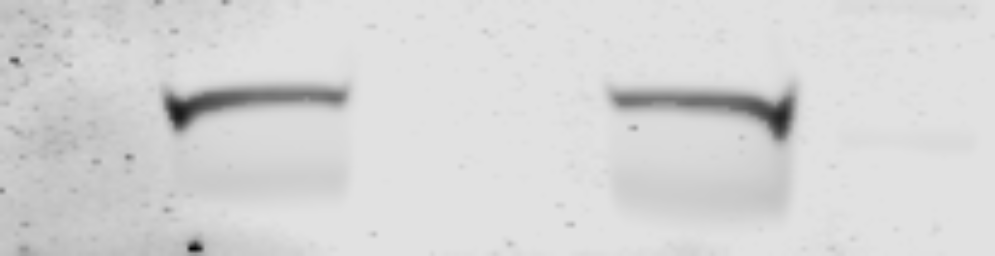

Supplement: Figure 3—source data 2. [file elife-104545-fig3-data2.zip › Figure 3, source data 2/p-Rb (S807_811).png]

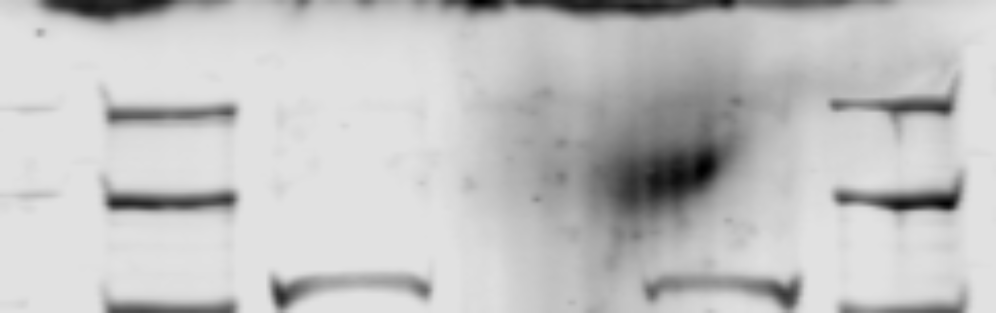

Supplement: Figure 3—source data 2. [file elife-104545-fig3-data2.zip › Figure 3, source data 2/t-Rb.png]

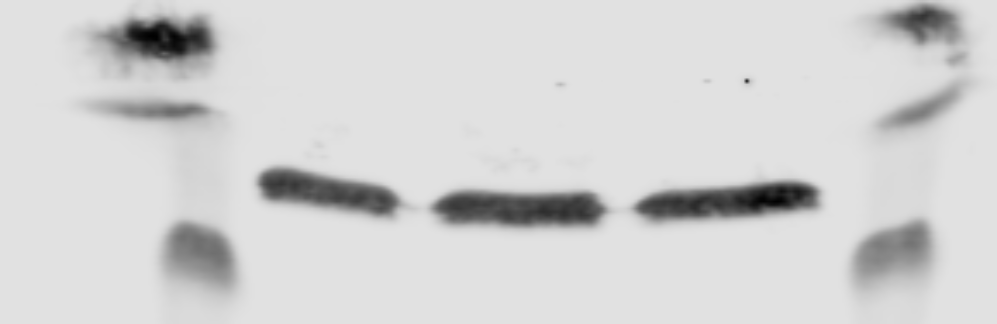

Supplement: Figure 3—figure supplement 1—source data 2. [file elife-104545-fig3-figsupp1-data2.zip › Figure 3, figure supplement 1, source data 2/b-actin.png]

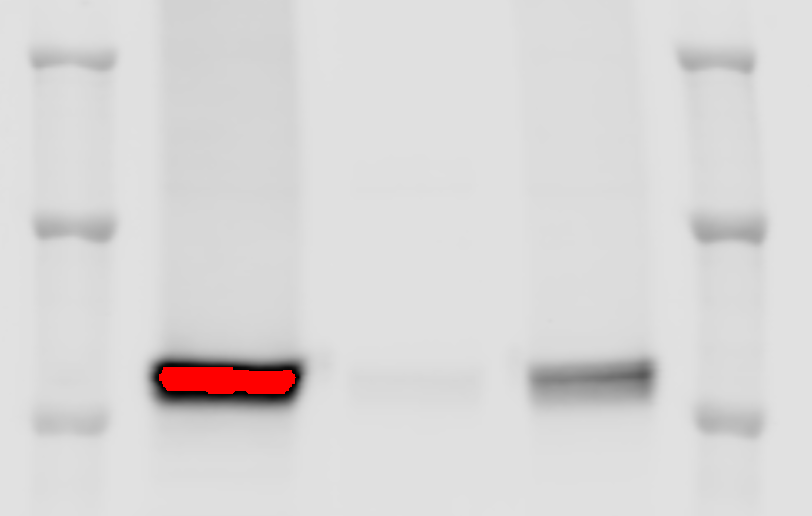

Supplement: Figure 3—figure supplement 1—source data 2. [file elife-104545-fig3-figsupp1-data2.zip › Figure 3, figure supplement 1, source data 2/t-Rb saturation highlighted.png]

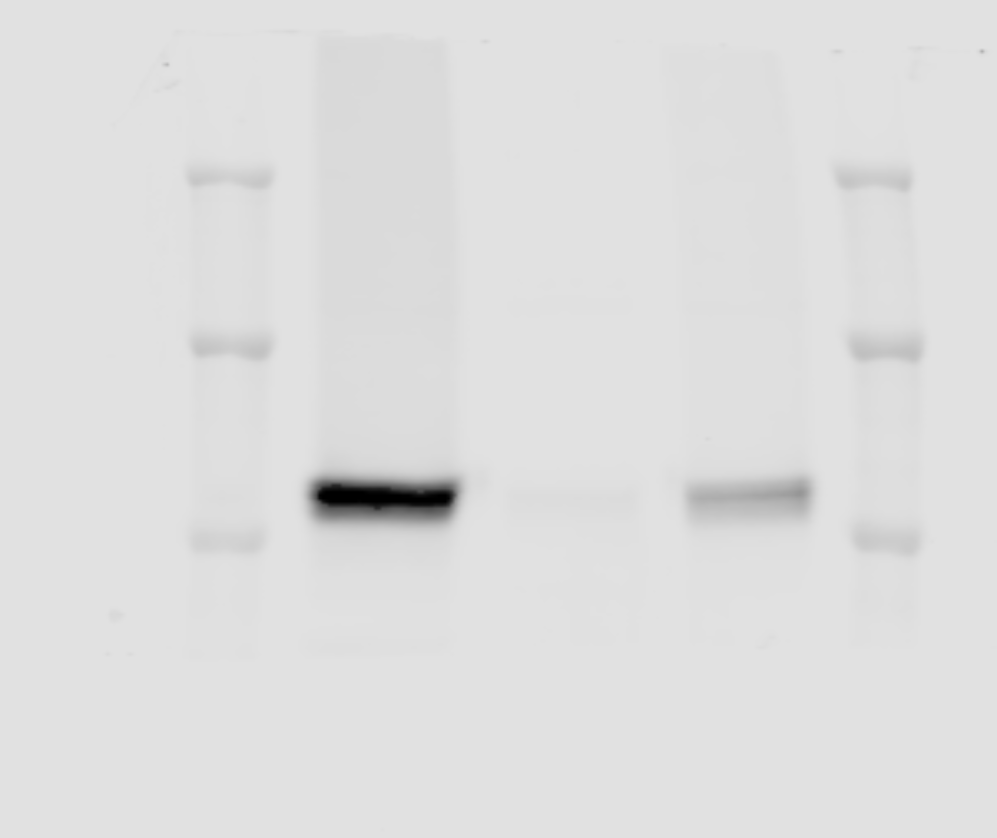

Supplement: Figure 3—figure supplement 1—source data 2. [file elife-104545-fig3-figsupp1-data2.zip › Figure 3, figure supplement 1, source data 2/t-Rb.png]

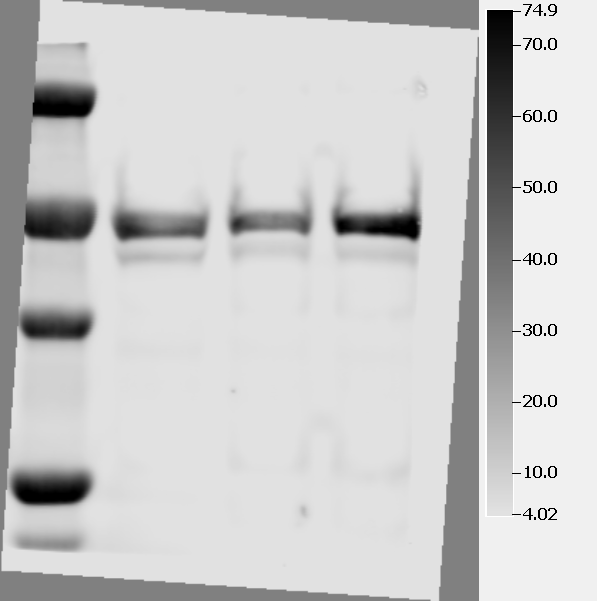

Supplement: Figure 7—figure supplement 1—source data 2. [file elife-104545-fig7-figsupp1-data2.zip › Figure 7, figure supplement 1, source data 2/Cyclin A.png]

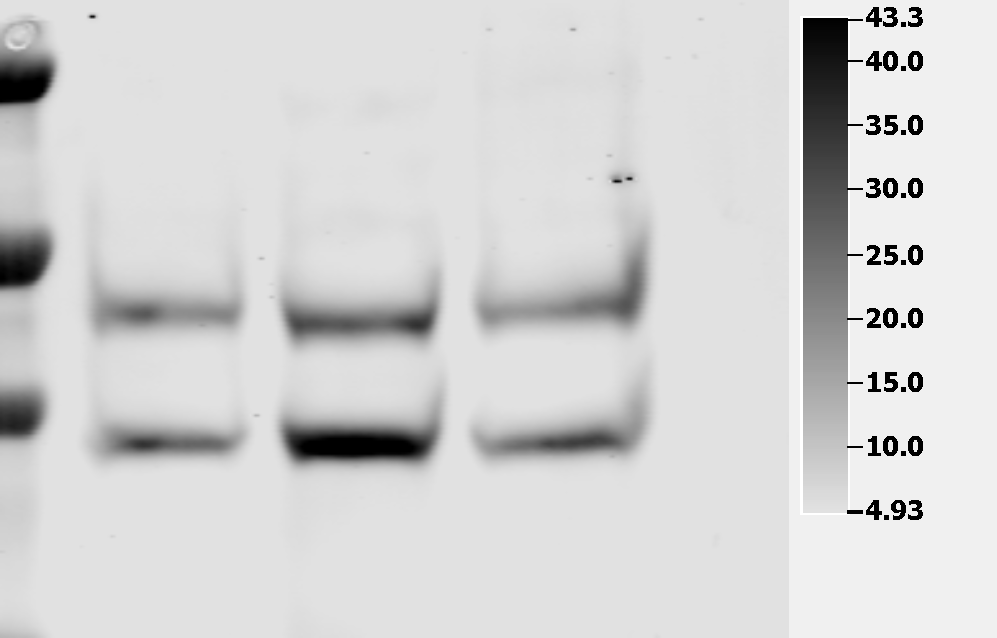

Supplement: Figure 7—figure supplement 1—source data 2. [file elife-104545-fig7-figsupp1-data2.zip › Figure 7, figure supplement 1, source data 2/Cyclin E.png]

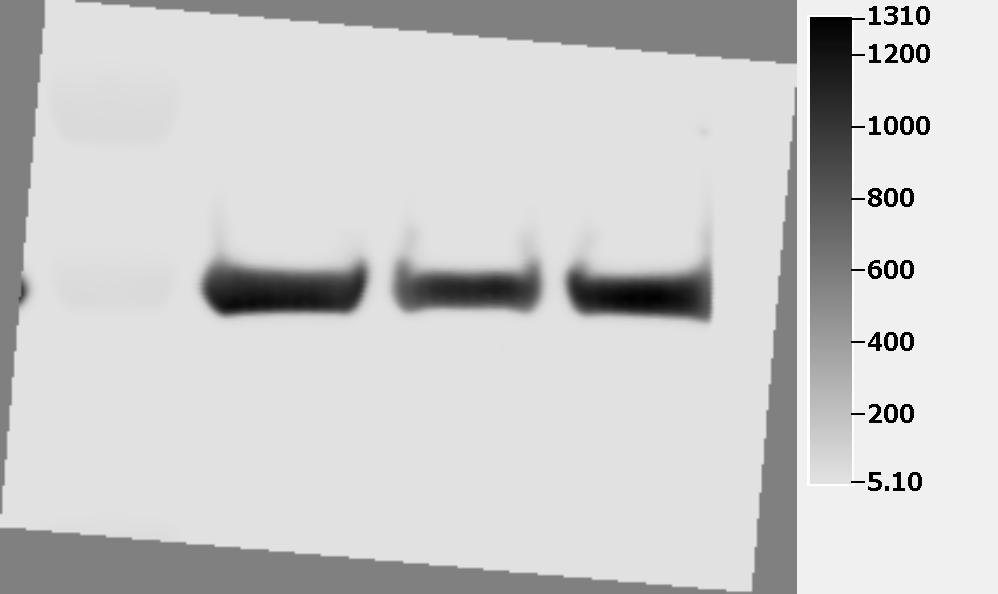

Supplement: Figure 7—figure supplement 1—source data 2. [file elife-104545-fig7-figsupp1-data2.zip › Figure 7, figure supplement 1, source data 2/GAPDH_Cyclin A blot.png]

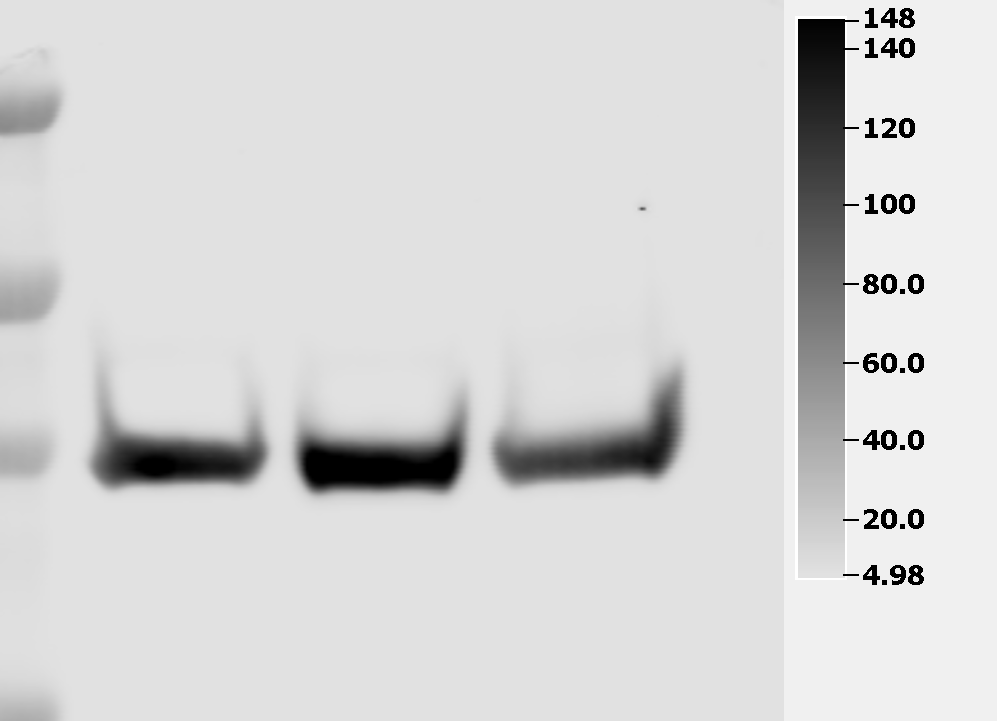

Supplement: Figure 7—figure supplement 1—source data 2. [file elife-104545-fig7-figsupp1-data2.zip › Figure 7, figure supplement 1, source data 2/GAPDH_Cyclin E blot.png]
