# Supplementary material for: Therapeutic benefits of maintaining CDK4/6 inhibitors and incorporating CDK2 inhibitors beyond progression in breast cancer
Source: eLife. 2025 Dec 29;14:RP104545. doi: 10.7554/eLife.104545 (PMC12747521; doi:10.7554/eLife.104545)
Supplement: Figure 3—source data 1. [file elife-104545-fig3-data1.zip › Figure 3, source data 1/Figure 3, source data 1.pdf]

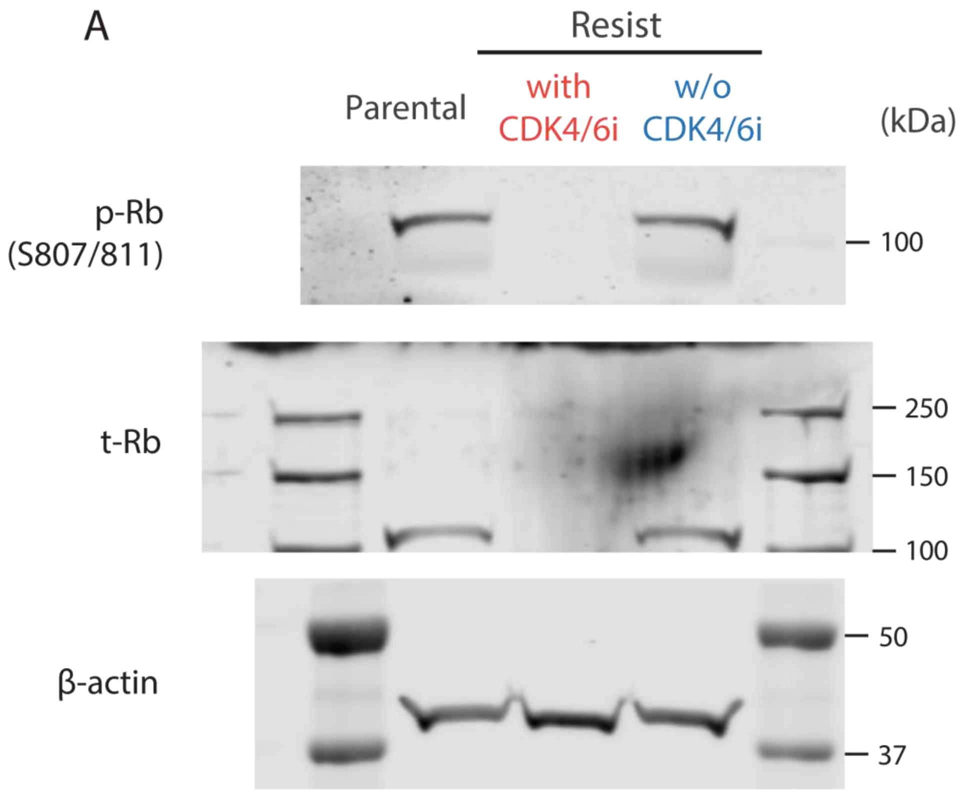

Figure 3, source data 1. Original membranes corresponding to Figure 3A. Immunoblot of phosphorylated Rb (S807/811; p-Rb), total Rb (t-Rb), and β-actin protein levels in MCF-7 cells. Drug-resistant cells were harvested two weeks after drug withdrawal. Precision plus protein standards were used and molecular weights indicated.
