# Supplementary material for: Therapeutic benefits of maintaining CDK4/6 inhibitors and incorporating CDK2 inhibitors beyond progression in breast cancer
Source: eLife. 2025 Dec 29;14:RP104545. doi: 10.7554/eLife.104545 (PMC12747521; doi:10.7554/eLife.104545)
Supplement: Figure 3—figure supplement 1—source data 1. [file elife-104545-fig3-figsupp1-data1.zip › Figure 3, figure supplement 1, source data 1/Figure 3, figure supplement 1, source data 1.pdf]

A

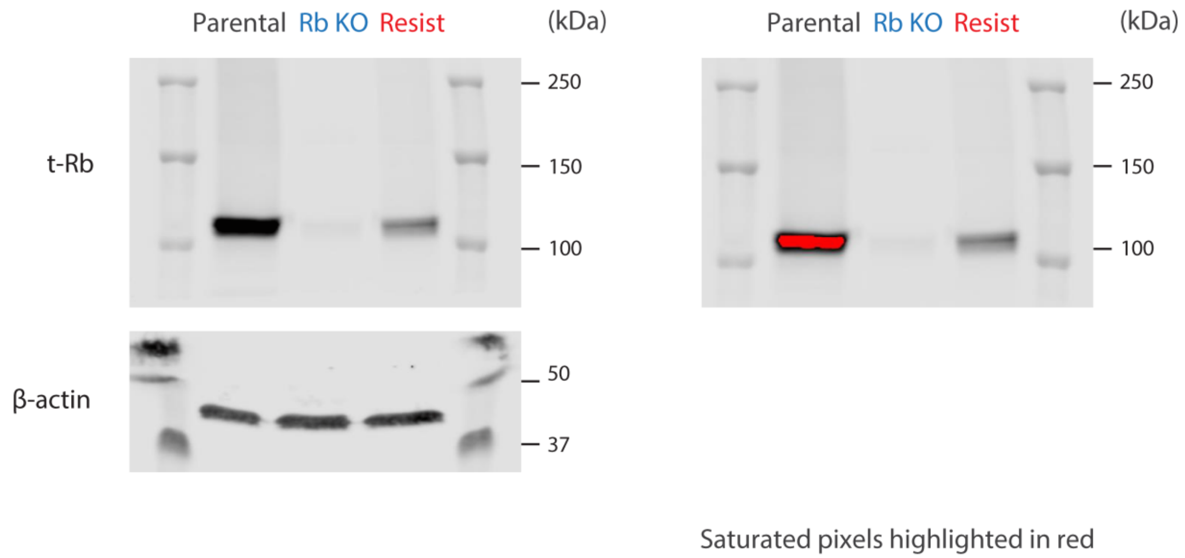

Figure 3, figure supplement 1, source data 1. Original membranes corresponding to Figure 3 – figure supplement 2A. Immunoblot showing total Rb and β-actin in WT, Rb-KO, and palbociclib-resistant cells. Precision plus protein standards were used and molecular weights indicated. The image on the right shows saturated pixels highlighted in red.
