# Supplementary material for: Therapeutic benefits of maintaining CDK4/6 inhibitors and incorporating CDK2 inhibitors beyond progression in breast cancer
Source: eLife. 2025 Dec 29;14:RP104545. doi: 10.7554/eLife.104545 (PMC12747521; doi:10.7554/eLife.104545)
Supplement: Figure 7—figure supplement 1—source data 1. [file elife-104545-fig7-figsupp1-data1.zip › Figure 7, figure supplement 1, source data 1/Figure 7, figure supplement 1, source data 1.pdf]

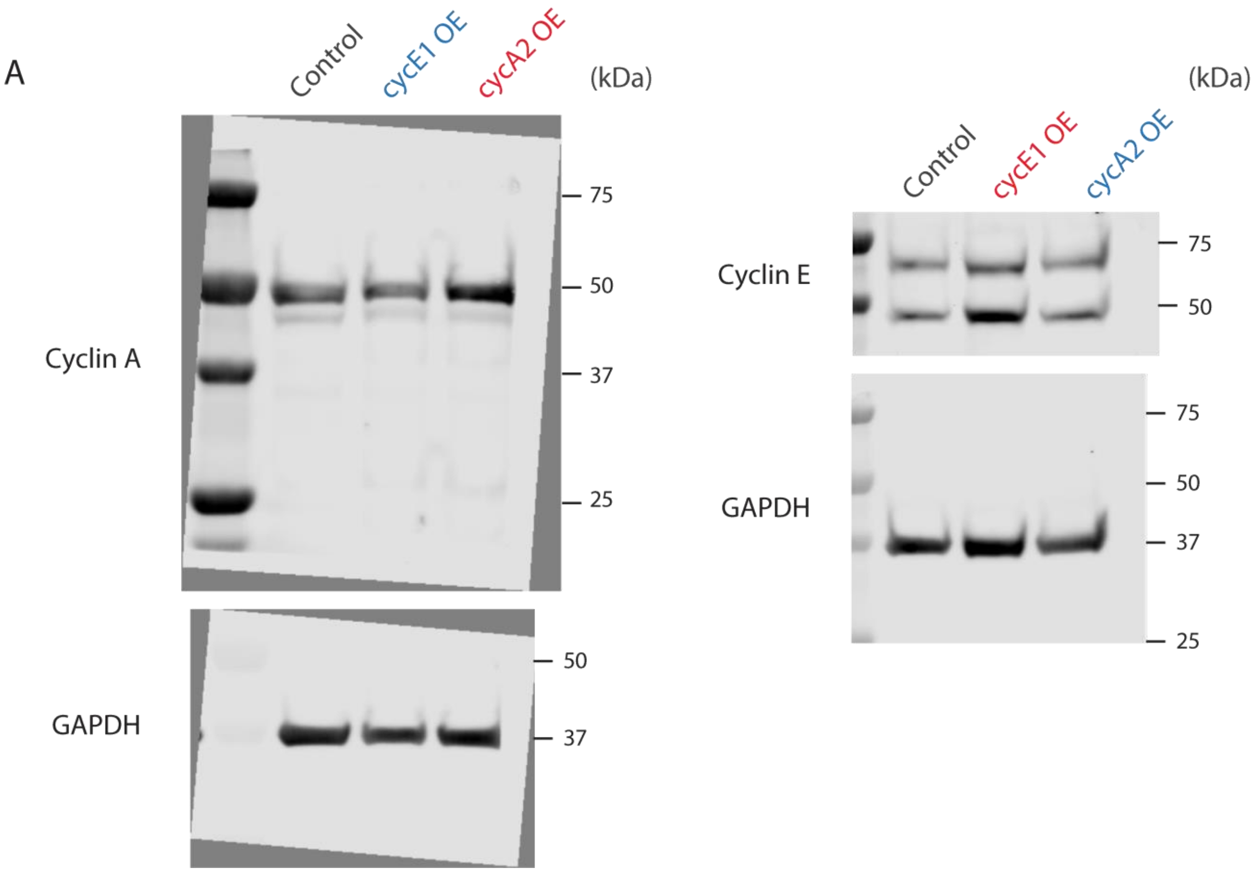

Figure 7, figure supplement 1, source data 1. Original membranes corresponding to Figure 7 – figure supplement 1A. Immunoblot showing the expression of GAPDH with cyclin A (left) or cyclin E (right) in MCF-7 cells with or without cyclin E1 or A2 overexpression. Cells were treated with doxycycline (500 nM) for 24 hr. Precision plus protein standards were used and molecular weights indicated.
