# Supplementary material for: Therapeutic benefits of maintaining CDK4/6 inhibitors and incorporating CDK2 inhibitors beyond progression in breast cancer
Source: eLife. 2025 Dec 29;14:RP104545. doi: 10.7554/eLife.104545 (PMC12747521; doi:10.7554/eLife.104545)
Supplement: Supplementary file 1. [file elife-104545-supp1.docx]

**Supplementary File 1, Statistics Summary**

| **Figure** | **Statistical test** | **P value** | **Test Statistics** |
| --- | --- | --- | --- |
| Fig. 1A | Two way ANOVA with Tukey’s post hoc multiple comparisons | **MCF-7**  Naïve-resistant: P = <0.0001 Naïve-withdrawn: P = 0.2072 Resistant-withdrawn: P<0.0001  **T47D**  Naïve-resistant: P = <0.0001 Naïve-withdrawn: P = 0.2897 Resistant-withdrawn: P<0.0001  **CAMA1**  Naïve-resistant: P = <0.0001 Naïve-withdrawn: P = 0.1988 Resistant-withdrawn: P<0.0001  **MDAMB231**  Naïve-resistant: P = <0.0001 Naïve-withdrawn: P = 0.4579 Resistant-withdrawn: P<0.0001 | **MCF-7**  Naïve-resistant: 95% CI = [488958, 667232]  Naïve-withdrawn: 95% CI = [-25780, 152494]  Resistant-withdrawn: 95% CI = [-603875, -425601]  **T47D**  Naïve-resistant: 95% CI = [13249, 18180]  Naïve-withdrawn: 95% CI =[  -4013, 917.9]  Resistant-withdrawn: 95% CI =[  -19727, -14796]  **CAMA1**  Naïve-resistant: 95% CI = [  677542, 838458]  Naïve-withdrawn: 95% CI =[  -22482, 138434]  Resistant-withdrawn: 95% CI  -780482, -619566]  **MDAMB231**  Naïve-resistant: 95% CI = [  1640031, 2163064]  Naïve-withdrawn: 95% CI  -131993, 391040]  Resistant-withdrawn: 95% CI  -2033540, -1510507] |
| Fig. 4C | Two way ANOVA with Tukey’s post hoc multiple comparisons | Discontinue-palbociclib: P = <0.0001  Discontinue-abemaciclib: P = 0.0001  Discontinue-ribociclib: P = <0.0001  Palbociclib-abemaciclib: P = <0.0001  Palbociclib-ribociclib: P = <0.0001  Abemaciclib-ribociclib: P =0.3456 | Discontinue-palbociclib: 95% CI = [  91.07, 150.9]  Discontinue-abemaciclib: 95% CI = [163.5, 223.3]  Discontinue-ribociclib: 95% CI = [182.7, 242.5]  Palbociclib-abemaciclib: 95% CI = [42.54, 102.3]  Palbociclib-ribociclib: 95% CI = -=  [61.72, 121.5]  Abemaciclib-ribociclib: 95% CI = [  -10.72, 49.08] |
| Fig. 4E | One way ANOVA with Tukey’s post hoc analysis | Discontinue-palbociclib: P = <0.0018  Discontinue-abemaciclib: P = 0.0001  Discontinue-ribociclib: P = <0.0001  Palbociclib-abemaciclib: P = 0.5428  Palbociclib-ribociclib: P = 0.3780  Abemaciclib-ribociclib: P = 0.9904 | Discontinue-palbociclib: 95% CI = [149.2, 666.4]  Discontinue-abemaciclib: 95% CI = [271.8, 789.0]  Discontinue-ribociclib: 95% CI = [298.8, 816.0]  Palbociclib-abemaciclib: 95% CI =  [-136.0, 381.2]  Palbociclib-ribociclib: 95% CI = [-109.0, 408.2]  Abemaciclib-ribociclib: 95% CI = [-231.6, 285.6] |
| Fig. 4F | One way ANOVA with Tukey’s post hoc analysis | Discontinue-palbociclib: P = <0.0001  Discontinue-abemaciclib: P = <0.0001  Discontinue-ribociclib: P = <0.0001  Palbociclib-abemaciclib: P = 0.3728  Palbociclib-ribociclib: P = 0.4153  Abemaciclib-ribociclib: P = 0.9998 | Discontinue-palbociclib: 95% CI = [28.2, 65]  Discontinue-abemaciclib: 95% CI = [38.91, 75.71]  Discontinue-ribociclib: 95% CI = [38.38, 75.18]  Palbociclib-abemaciclib: 95% CI = [-7.691, 29.11]  Palbociclib-ribociclib: 95% CI = [-8.222, 28.58]  Abemaciclib-ribociclib: 95% CI = [-18.93, 17.87] |
| Fig. 5A | One way ANOVA with Tukey’s post hoc analysis | MCF-7-CAMA-1: P = 0.0002  MCF-7-T47D: P = 0.0002  CAMA-1-T47D: P = 0.9921 | MCF-7-CAMA-1: 95% CI = [  26344, 52296]  MCF-7-T47D 95% CI = [  26850, 52803]  CAMA-1-T47D 95% CI = [  -12470, 13482] |
| Fig. 5B | Two way ANOVA with Tukey’s post hoc multiple comparisons | **MCF-7**  Palbo + Fulv-Palbo: P = <0.0001  Palbo + Fulv-Fulv: P = <0.0001  Palbo-Fulv: P = <0.0001  **CAMA-1**  Palbo + Fulv-Palbo: P = <0.0001  Palbo + Fulv-Fulv: P = <0.0001  Palbo-Fulv: P = <0.0001 | **MCF-7**  Palbo + Fulv-Palbo: 95% CI = [-449873, -243261]  Palbo + Fulv-Fulv: 95% CI = [-1319139, -1112527]  Palbo-Fulv: 95% CI = [-972573, -765961]  **CAMA-1**  Palbo + Fulv-Palbo: 95% CI =  [-620647, -388686]  Palbo + Fulv-Fulv: 95% CI =  [-1085897, -853936]  Palbo-Fulv: 95% CI =  -581230, -349270] |
| Fig. 6A | Two way ANOVA with Tukey’s post hoc multiple comparisons | **MCF-7**  Discontinue-Fulv d20: P = <0.0001  **CAMA-1**  Discontinue-Fulv d20: P =<0.0001 | **MCF-7**  Discontinue-Fulv: 95% CI =  [18688427, 24541573]  **CAMA-1**  Discontinue-Fulv: 95% CI = [52820806, 59962528] |
| Fig. 6B | Two way ANOVA with Tukey’s post hoc multiple comparisons | **MCF-7**  Fulv+palbo- Fulv+INX-315: P =<0.0001  Fulv+palbo-Fulv+palbo+INX315: P =<0.0001  Fulv+INX315-Fulv+palbo+INX-315: P = <0.0001  **CAMA-1**  Fulv+palbo- Fulv+INX-315: P =<0.0001  Fulv+palbo-Fulv+palbo+INX315: P =<0.0001  Fulv+INX315-Fulv+palbo+INX-315: P = <0.0001 | **MCF-7**  Fulv+palbo- Fulv+INX-315: 95% CI = [-169373, -95127]  Fulv+palbo-Fulv+palbo+INX315: 95% CI = [59405, 133651]  Fulv+INX315-Fulv+palbo+INX-315 95% CI = [191655, 265901]  **CAMA-1**  Fulv+palbo- Fulv+INX-315: 95% CI = [-140925, -83436]  Fulv+palbo-Fulv+palbo+INX315: 95% CI = [100811, 158300]  Fulv+INX315-Fulv+palbo+INX-315 95% CI = [212992, 270481] |
| Fig. 6C | Unpaired t-test | **MCF-7**  Discontinue-Fulv: P = 0.0009  Discontinue- Palbo+Fulv: P = 0.0003  Discontinue- INX-315+Fulv: P = 0.0003  Discontinue- Palbo+Fulv+INX-315: P = 0.0003  Fulv-Palbo+Fulv: P = 0.0070  Fulv- INX-315+Fulv: P = 0.0095  Fulv- Palbo+INX-315+Fulv: P = 0.0055  Fulv+palbo- Fulv+INX-315: P = 0.0019  Fulv+palbo-Fulv+palbo+INX315: P = 0.0099  Fulv+INX315-Fulv+palbo+INX-315: P = 0.0007  **CAMA-1**  Discontinue-Fulv: P =<0.0001  Discontinue- Palbo+Fulv: P = <0.0001  Discontinue- INX-315+Fulv: P = <0.0001  Discontinue- Palbo+Fulv+INX-315: P = <0.0001  Fulv-Palbo+Fulv: P = <0.0001  Fulv- INX-315+Fulv: P = <0.0001  Fulv- Palbo+INX-315+Fulv: P = <0.0001  Fulv+palbo- Fulv+INX-315: P = 0.0103  Fulv+palbo-Fulv+palbo+INX315: P = 0.0008  Fulv+INX315-Fulv+palbo+INX-315: P = 0.0001 | **MCF-7**  Discontinue-Fulv: 95% CI = [-28336715, -14893285]  Discontinue- Palbo+Fulv: 95% CI = [-32424563, -19927104]  Discontinue- INX-315+Fulv: 95% CI = [-32044284, -19545716]  Discontinue- Palbo+Fulv+INX-315: 95% CI = [-32730260, -20229740]  Fulv-Palbo+Fulv: 95% CI = [-7040574, -2081093]  Fulv- INX-315+Fulv: 95% CI = [-6661138, -1698862]  Fulv- Palbo+INX-315+Fulv: 95% CI = [-7348596, -2381404]  Fulv+palbo- Fulv+INX-315: 95% CI = [235217, 526450]  Fulv+palbo-Fulv+palbo+INX315: 95% CI = [-486949, -121384]  Fulv+INX315-Fulv+palbo+INX-315 95% CI = [-885853, -484147]  **CAMA-1**  Discontinue-Fulv: 95% CI = [-64746062, -48037272]  Discontinue- Palbo+Fulv: 95% CI = [-69823809, -53196191]  Discontinue- INX-315+Fulv: 95% CI = [-69594695, -52966972]  Discontinue- Palbo+Fulv+INX-315: 95% CI = [-70176741, -53549926]  Fulv-Palbo+Fulv: 95% CI = [-5952002, -4284664]  Fulv- INX-315+Fulv: 95% CI = [-5723356, -4054978]  Fulv- Palbo+INX-315+Fulv: 95% CI = [-6301319, -4642014]  Fulv+palbo- Fulv+INX-315: 95% CI = [89959, 368374]  Fulv+palbo-Fulv+palbo+INX315: 95% CI = [-462102, -244565]  Fulv+INX315-Fulv+palbo+INX-315 95% CI = [-695185, -469815] |
| Fig 7F | Two way ANOVA with Tukey’s post hoc multiple comparisons | WT-cyclinE OE: P < 0.0001  WT-cyclinA OE: P < 0.0001  cyclinE OE-cyclinA OE: P < 0.0001 | WT-cyclinE OE 95% CI [-15391005, -10451661]  WT-cyclinA OE 95% CI [-8915339, -3975995]  cyclinE OE-cyclinA OE 85% CI [4005995, 8945339] |
| Fig 5 Supplement 1A | Two way ANOVA with Tukey’s post hoc multiple comparisons | Discontinued-Palbo: P = <0.0001  Discontinued-Palbo+cMyc: P <0.0001  Palbo-Palbo+cMyc: P = <0.0001 | Discontinued- Palbo: 95% CI = [500308, 663739]  Discontinued- Palbo+cMyc: 95% CI = [193284, 356716]  Palbo - Palbo+cMyc: 95% CI = [-388739, -225308] |
| Fig. 5 Supplement 2 B | Unpaired t-test | **MCF-7**  Discontinue-Palbo + Fulv: P = 0.0015  Discontinue-Palbo: P = 0.0018  Discontinue-Fulv: P = 0.0035  Palbo + Fulv-Palbo: P = 0.0003  Palbo + Fulv-Fulv: P = <0.0001  Palbo-Fulv: P = 0.0001  **CAMA-1**  Discontinue-Palbo + Fulv: P = <0.0001  Discontinue-Palbo: P = <0.0001  Discontinue-Fulv: P = <0.0001  Palbo + Fulv-Palbo: P = 0.0006  Palbo + Fulv-Fulv: P = <0.0001  Palbo-Fulv: P = 0.0019 | **MCF-7**  Discontinue-Palbo + Fulv: 95% CI = [-41453483, -19651851]  Discontinue-Palbo: 95% CI = [-39769440, -17953893]  Discontinue-Fulv: 95% CI = [-35135066, -13283601]  Palbo + Fulv-Palbo: 95% CI = [1293157, 2088843]  Palbo + Fulv-Fulv: 95% CI = [5601455, 7085211]  Palbo-Fulv: 95% CI = [3814407 5490259]  **CAMA-1**  Discontinue-Palbo + Fulv: 95% CI = [-55078918, -41907082]  Discontinue-Palbo: 95% CI = [-52789882, -39556785]  Discontinue-Fulv: 95% CI = [-50346155, -37105845]  Palbo + Fulv-Palbo: 95% CI = [1673213, 2966121]  Palbo + Fulv-Fulv: 95% CI = [4084619, 5449381]  Palbo-Fulv: 95% CI = [1514574, 3380093] |
